# Effect of Cleaning After Silica-Coating on the Resin Bond Strength to Zirconia

**DOI:** 10.1590/0103-644020267056

**Published:** 2026-10-19

**Authors:** Kétlin Fagundes Teixeira, Fernanda Cocco, Renatta Wrasse Temp, Gabriel Bauken, Pablo Machado Soares, Gabriel Kalil Rocha Pereira, Ana Carolina Cadore-Rodrigues, Luiz Felipe Valandro

**Affiliations:** 1 Program in Oral Science, Faculty of Dentistry, Federal University of Santa Maria (UFSM), Santa Maria, RS, Brazil

**Keywords:** Aging, Cleaning methods, Microshear bond strength, Silica-coated alumina particles, Surface treatment

## Abstract

This study assessed whether the cleaning protocol of the bonding surface of translucent zirconia, after silica-coating by air-abrasive particles, affects its resin bond strength before and after long-term aging. Zirconia slices (15×15×2 mm) (IPS e.max ZirCAD MT, Ivoclar) were air-abraded with silica-coated alumina particles, and then assigned to four cleaning protocol groups: air-jet only (AJ), air/water spray (AWS), ultrasonic bath with alcohol (UB-A), or ultrasonic bath with distilled water (UB-W). All surfaces received a primer (Monobond Plus, Ivoclar) followed by bonding the resin cement cylinders (Variolink Esthetic DC, Ivoclar; n=15), to be tested in microshear bond strength (µSBS) after 24 h of water storage (baseline) or after aging (90 days of water storage + 20,000 thermocycles, 5- 55 °C). Surface topography, elemental composition, and failure modes were also performed. Two-way ANOVA showed no significant effects of cleaning protocol (F=0.383, p=0.765), aging (F=3.196, p=0.077), or their interaction (F=0.443, p=0.723) on bond strength, although aged groups exhibited a higher incidence of pre-test failures. SEM revealed similar surface morphology among all groups, characteristic of silica-coated air abrasion. EDS detected residual silica in the AJ (0.8%) and AWS (0.13%) groups, whereas UB-A showed lower silica content (0.07%), and no silica was detected in UB-W. Despite these chemical differences, all cleaning protocols resulted in comparable bonding performance, with adhesive and cohesive failures observed across all groups. These findings indicate that cleaning protocol does not significantly affect zirconia-resin bond strength, and simpler approaches, such as air or air/water jets, are effective for maintaining bond strength after aging.

## Resumo

O presente estudo avaliou se a limpeza da superfície de união de restaurações em uma zircônia translúcida após o jateamento com óxido de alumínio revestido por sílica influencia a resistência de união ao cimento resinoso, antes e após o envelhecimento artificial. Lâminas de zircônia (15 × 15 × 2 mm) foram confeccionadas a partir de IPS e.max ZirCAD MT (Ivoclar AG) e submetidas ao jateamento com alumina revestida por sílica. Em seguida, os espécimes foram distribuídos em quatro grupos de acordo com o protocolo de limpeza: jato de ar apenas (AJ), spray ar/água (AWS), banho ultrassônico com álcool (UB-A) e banho ultrassônico com água destilada (UB-W). Todas as superfícies receberam a aplicação ativa de um primer (Monobond Plus, Ivoclar AG) por 20 s, seguida de secagem suave com ar. Cilindros de cimento resinoso (Variolink Esthetic DC, Ivoclar AG; n = 15) foram cimentados às superfícies de zircônia e submetidos ao ensaio de resistência de união ao microcisalhamento (µSBS) após 24 h de armazenamento em água (baseline) e após envelhecimento artificial, consistindo em 90 dias de armazenamento em água e 20.000 ciclos térmicos (5-55 °C). A topografia superficial (MEV, n= 1), a composição elementar (EDS, n= 1) e os modos de falha também foram analisados. A ANOVA de dois fatores não revelou efeito significativo do método de limpeza (F= 0,383; p= 0,765), do envelhecimento (F= 3,196; p= 0,077) ou da interação entre esses fatores (F = 0,443; p= 0,723) sobre a resistência de união, embora os grupos envelhecidos tenham apresentado maior incidência de falhas pré-teste. A análise em MEV evidenciou morfologia superficial semelhante entre todos os grupos, característica do jateamento com sílica. O EDS detectou sílica residual nos grupos AJ (0,8%) e AWS (0,13%), enquanto o grupo UB-A apresentou menor teor de silício (0,07%) e nenhuma sílica foi detectada no grupo UB-W. Apesar dessas diferenças químicas, todos os protocolos de limpeza resultaram em desempenho de união comparável, com ocorrência de falhas adesivas e coesivas em todos os grupos. Esses achados indicam que o método de limpeza não afeta significativamente a resistência de união entre uma ircônia translúcida e o cimento resinoso, e que abordagens mais simples, como jato de ar ou ar/água, são eficazes para manter o desempenho adesivo após o envelhecimento.

**Figure:**
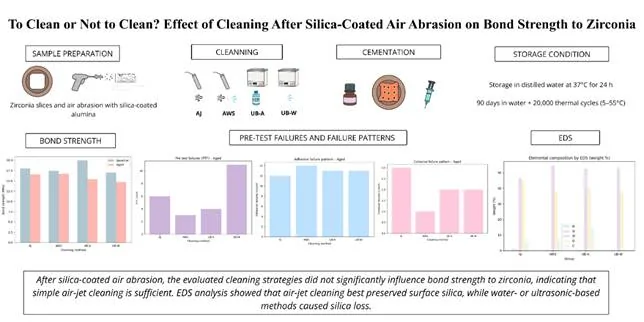

## Introduction

Ceramic restorations have become an excellent rehabilitative option in contemporary dentistry due to the intrinsic properties of these materials ^1^ ^,^ ^2^ ^,^ ^3^. Among the available ceramic options, yttria-stabilized zirconia (Y-SZ) stands out for its superior mechanical properties compared with glass ceramics, combined with excellent biocompatibility ^4^ ^,^ ^5^. These materials have been recognized as major contributors in the pursuit of highly durable, predictable, and aesthetically pleasing restorations ^5^ ^,^ ^6^ ^,^ ^7^, which aligns with current literature, in which the predictability of mechanical performance is highlighted as a key factor ^8^ ^,^ ^9^. 

Despite its excellent mechanical properties, zirconia exhibits inherent limitations related to its microstructural composition when it comes to bonding ^10^ ^,^ ^11^. It is a polycrystalline material composed primarily of zirconium dioxide (ZrO₂) stabilized with yttrium oxide (Y₂O₃), with small additions of aluminum oxide (Al₂O₃) to refine the grain structure ^2^ ^,^ ^6^ ^,^ ^12^ ^,^ ^13^. In this sense, the absence of a glassy phase and consequently of silica prevents the use of hydrofluoric acid etching, and silane coupling becomes ineffective, limiting both micromechanical and chemical bonding to resin cements ^14^ ^,^ ^15^ ^,^ ^16^. Thus, zirconia depends on different topographic modifications achieved through surface treatments such as air abrasion with abrasive particles ^17^ ^,^ ^18^. These interventions aim to increase surface energy and create micro-irregularities that enhance the micromechanical retention of the resin cement ^18^ ^,^ ^19^ ^,^ ^20^.

With the aim of simultaneously enhancing both the chemical and mechanical bonding to zirconia, silica-coated alumina particles were introduced as an alternative to conventional aluminum oxide air abrasion ^21^ ^,^ ^22^. Studies have reported increased bond strength and satisfactory adhesive performance with this treatment ^23^ ^,^ ^24^. The mechanism of action is based on the projection of silica-coated alumina particles under high pressure against the zirconia surface ^24^ ^,^ ^25^. The impact of these particles promotes controlled surface erosion, creating micro-irregularities that enhance micromechanical retention while simultaneously depositing a thin silica layer onto the substrate ^15^ ^,^ ^26^ ^,^ ^27^. This silica-coated layer enables the formation of chemical bonds with silane agents, which in turn establish siloxane bonds between the ceramic surface and the resin matrix of the cement ^28^ ^,^ ^29^ ^,^ ^30^. Thus, silica-coating creates a hybrid interface in which micromechanical and chemical mechanisms act synergistically, promoting satisfactory bonding ^22^.

However, it is important to emphasize that bonding is one of the main determinants of the longevity and predictability of ceramic restorations ^29^ ^,^ ^31^. For the adhesive process to be successful, it is essential that all steps of the surface treatment are standardized and well understood ^29^. The cleaning step plays a critical role, as the presence of debris, abrasive residues, or organic contaminants can reduce the surface energy of zirconia, impair wettability, and compromise the formation of stable chemical bonds between the silane and the resin cement ^30^ ^,^ ^31^ ^,^ ^32^. In this sense, establishing a standardized cleaning protocol is fundamental to ensure consistency and reliability in bonding outcomes, especially considering that variations in surface condition may directly influence the effectiveness of the chemical interaction.

In this context, an important question arises: after air abrasion with silica-coated alumina particles, what is the most effective protocol for cleaning the ceramic surface? According to the manufacturer’s recommendations, air-jet cleaning should be performed after the silica-coated procedure to remove loosely attached particles and superficial debris without disrupting the silica layer. This method is considered conservative and aims to preserve the reactive surface created by tribochemical treatment. Nevertheless, some studies describe the use of an ultrasonic bath following air abrasion ^33^ ^,^ ^34^, as it may enhance the removal of residual contaminants through vibration. Despite this potential advantage, its effectiveness remains controversial, as part of the literature suggests that ultrasonic cleaning may promote partial displacement of the deposited silica layer ^35^ ^,^ ^36^ thereby affecting its chemical interaction with silane. Conversely, other findings indicate that additional cleaning steps, including an ultrasonic bath or even air-water spray, may negatively impact bond strength when compared to no cleaning ^37^, possibly due to disruption or instability of the silica-coated layer, reinforcing the need for a well-defined and evidence-based protocol.

Furthermore, zirconia has undergone significant advancements in its forms of presentation, mainly through modifications in the concentration of stabilizing oxides ^38^ ^,^ ^39^ ^,^ ^40^ ^,^ ^41^. Conventional zirconia containing 3 mol% yttria (3Y-TZP) has been progressively replaced by compositions with 4-6 mol% yttria, known as translucent zirconia, such as 4’-YSZ, developed to minimize one of its main limitations: esthetics ^6^ ^,^ ^41^ ^,^ ^42^ ^,^ ^43^. This modification leads to changes in the microstructural arrangement, with increased cubic phase content ^44^ ^,^ ^45^, enhancing light transmission and resulting in a more translucent zirconia ^6^ ^,^ ^42^. However, this also leads to a reduced contribution of the transformation toughening mechanism ^41^. Although zirconia has been extensively investigated over the past decades, most of the available evidence is based on conventional 3Y-TZP materials. In contrast, the present study focuses on translucent zirconia.

Thus, despite its clear clinical relevance, up to now, few studies in the current literature have compared the effect of different cleaning protocols on the resin bond strength to translucent zirconia, highlighting a gap in knowledge that still requires further investigation. Therefore, the present study aimed to assess whether the cleaning protocol of the bonding surface of translucent zirconia after air abrasion with silica-coated alumina particles affects its bond strength to resin cement before and after long-term aging. The null hypothesis of the present study is that the cleaning protocol would not affect the bond strength between zirconia and resin cement.

## Materials and methods

### Materials and study design

Four distinct cleaning protocols were considered: air-jet only (AJ), air/water spray (AWS), ultrasonic bath with alcohol (UB-A), and ultrasonic bath with distilled water (UB-W). Additionally, the specimens were subjected to two storage conditions: no aging (baseline) and long-term aging through 90 days of water storage and 20,000 thermocycles (5-55 °C).

A pilot study considering preliminary mean and standard deviation values from the most discrepant groups in the baseline condition (30.69 ± 1.98 MPa; 13.20 ± 14.09 MPa) was conducted to calculate the required sample size using G*Power software (Version 3.1.9.6, Franz Faul, Universität Kiel, Kiel, Germany). This analysis considered a significance level (alpha, α) of 0.05, a statistical power of 95%, and an effect size (f) of 1.74, indicating a minimum requirement of 10 specimens (cement cylinders) per group. However, to increase the reliability and robustness of the results, 15 specimens per group were used. Zirconia slices were prepared per group, and 10 cement cylinders were cemented on each slab. This ensured that half of the specimens could be tested at baseline and the other half after aging, accounting for potential pre-test failures (PTF) during aging and securing a minimum of n= 15 per group. Pre-test failure specimens were discarded and replaced in the analysis, and cohesive failures were included in the statistical evaluation. Additional analyses included surface topography and failure mode assessment using scanning electron microscopy (SEM) and elemental composition by energy-dispersive X-ray spectroscopy (EDS).

A detailed description of all materials used in the study, commercial names, manufacturers, chemical compositions, and batch numbers, is provided in Table 1. The study design, including all factors, is summarized in Box 1. Figure 1 presents a flowchart illustrating the preparation steps of the specimens used in this study.

### Zirconia slices preparation

The zirconia slices were prepared from pre-sintered zirconia blocks (ZirCAD MT, Ivoclar AG, Schaan, Liechtenstein) following a standardized laboratory protocol. Initially, the blocks were sectioned into larger squares (approximately 20 mm × 20 mm) using a benchtop motor with a handpiece and diamond disc to allow proper mounting on a precision cutting machine. Subsequently, the squares were reduced to their final dimensions using a precision cutting machine (IsoMet 1000, Buehler, Lake Bluff, IL, USA) under water cooling. 

The cut surfaces were preliminarily polished with 600-grit silicon carbide (SiC) paper on a polishing machine (EcoMet/AutoMet 250, Buehler) to remove cutting marks. Final polishing was performed under water using a sequence of SiC papers (600, 1200, and 2500 grit) to achieve a smooth and uniform surface. The specimens were then sintered in a dedicated furnace (Vita Zyrcomat 6000 MS, Vita Zahnfabrik, Bad Säckingen, Baden-Württemberg, Germany) following the manufacturer’s recommended sintering cycle (heating to 900 °C at 10 °C/min, holding for 30 minutes, heating to 1500 °C at 3.3 °C/min, holding for 120 minutes, cooling to 900 °C at 10 °C/min, cooling to 300 °C at 8.3 °C/min, and turning off the furnace after reaching 300 °C). The final dimensions of the zirconia slices were 15 mm × 15 mm × 2 mm.

Subsequently, the zirconia slices were positioned inside polyvinyl chloride (PVC) cylinders (25 × 20 mm), and a self-curing acrylic resin (JET Clássico, Campo Limpo Paulista, SP, Brazil) was poured without contacting the bonding surface, which was kept exposed for the bonding procedures.

**Table 1:** Description of the materials used in the study, including their trade names, manufacturers, main compositions (in weight %), and batch numbers.

Material	Trade market and manufacturer	Main composition (% in weight)	Batch Number
Zirconia	IPS e.max ZirCAD MT, Ivoclar AG	ZrO2 (86.0-93.5 wt%) Y2O3 (6.5 % - 8.0 wt%) HfO2 (≤5.0 wt%) Al2O3 (≤1.0 wt%) and other oxides (≤1 0.0 wt%)	X39900
Silica-coated aluminum oxide particles	Cojet Sand, 3M Espe	30 µm silica-coated aluminum oxide particles	3628538
Silane coupling agent	Monobond Plus, Ivoclar AG	Alcohol solution of silane methacrylate, phosphoric acid methacrylate, and sulfide methacrylate	Z04Y1L
Resin cement	Variolink Esthetic DC, Ivoclar AG	Silicate glass, ytterbium trifluoride, highly dispersed silica, catalysts and stabilizer, pigments	Z05KP7

**Box 1:**
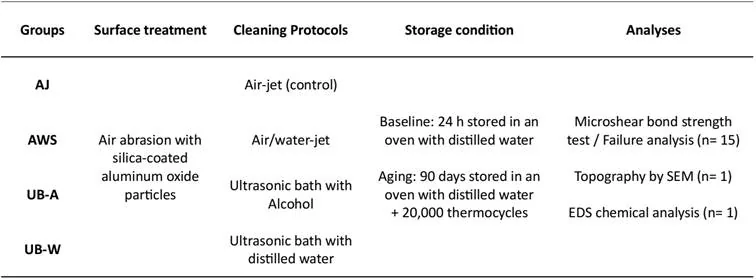
Study design including experimental groups, surface treatments, cleaning protocols, storage conditions, and analyses performed.

### Zirconia surface treatment

After preparation, the zirconia slices were cleaned using an ultrasonic cleaner (1440 D Odontobras, Ribeirão Preto, SP, Brazil) with distilled water for 5 min and treated with silica-coated air abrasion with 30 µm (Cojet ™ System, 3M ESPE) at a 10 mm distance, with a perpendicular angulation (90°) and 2.8 bar pressure for 10 sec.

**Figure 1:**
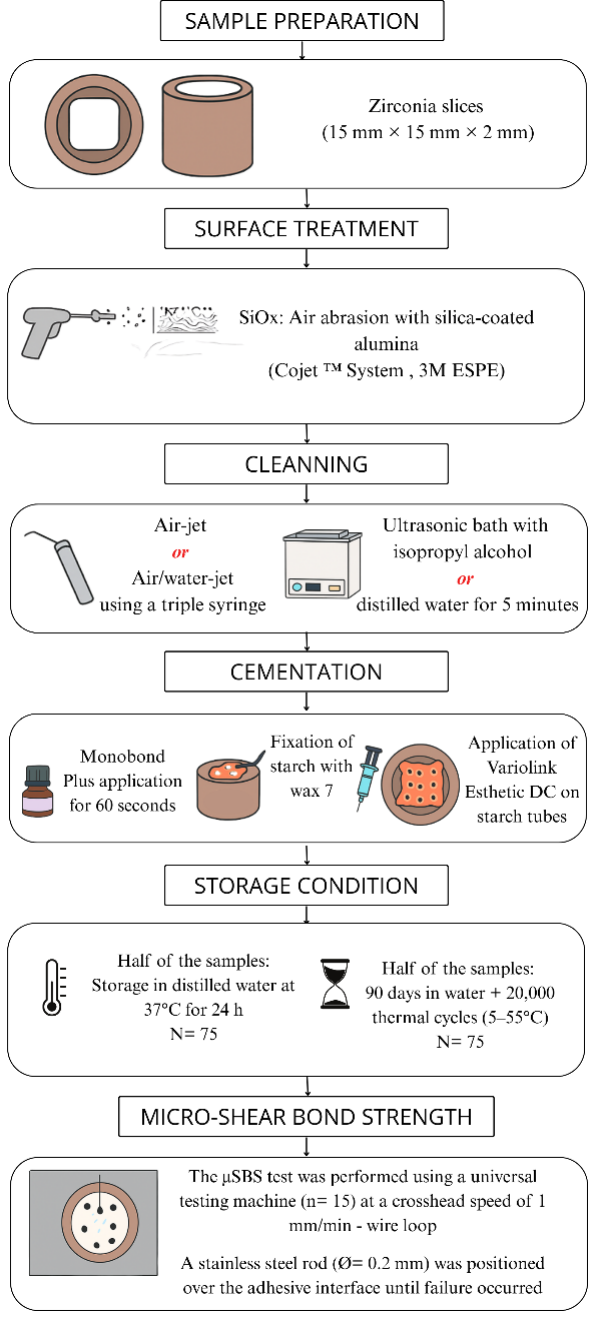
Flow chart illustrating the experimental study design.

### Cleaning protocols

After the surface treatment, the zirconia slices were subjected to different cleaning protocols:

### Air-jet

Continuous air stream delivered by a laboratory three-way syringe at 2.8 bar, positioned at a distance of 10 mm from the zirconia surface, for 10 seconds, being considered the control group.

### Air/water-jet

Combined air and water stream delivered by a laboratory three-way syringe at 2.8 bar, positioned at a distance of 10 mm from the zirconia surface, for 10 seconds, followed by drying with air-jet.

### Ultrasonic bath with alcohol

Zirconia slices were cleaned in an ultrasonic bath operating at 40 kHz with 99.8% isopropyl alcohol for 5 min and dried with air-jet.

### Ultrasonic bath with distilled water

Zirconia slices were cleaned in an ultrasonic bath operating at 40 kHz with distilled water for 5 min and dried with an air-jet.

### Luting procedure

After the cleaning protocols, the zirconia conditioned surface was coated with primer (Monobond Plus, Ivoclar AG, Schaan, Liechtenstein) for 20 s with active application, followed by a gentle air stream. On each slice, ten starch tubes (height = 1 mm, internal diameter = 1 mm) (Isabela, M. Dias Branco S.A. Indústria e Comércio de Alimentos, São Caetano do Sul, Brazil) were positioned on the bonding surface and fixed with sticky wax 7.

The resin cement (Variolink Automix, Ivoclar AG) was placed inside the tubes using a composite spatula and an endodontic plugger (#25, Dentsply Maillefer, Switzerland). Light-curing was then performed for 20 seconds (Radii-Cal, SDI, Bayswater, Australia; irradiance 1200 mW/cm²), strictly following the manufacturer’s instructions. After luting, the zirconia slices containing the cement cylinders were stored in distilled water at 37 °C for 24 h.

After removal of the starch tubes, the cement cylinders were carefully inspected, and any cement cylinders showing defects or interfacial bubbles were discarded. Half of the specimens (cement cylinders) from each plate were used for the microshear bond strength (µSBS) test after being stored in distilled water at 37 °C for 24 h (baseline). The remaining half underwent the aging protocol. For aging, the specimens were stored in distilled water at 37 °C for 90 days and subsequently subjected to 20,000 thermal cycles between 5 °C and 55 °C, with a dwell time of 30 seconds at each temperature and a 5-second transfer time between baths (Nova Ética, Vargem Grande do Sul, SP, Brazil).

### Microshear bond strength (µSBS) test

The zirconia slices were positioned in a fixation device attached to a universal testing machine (EMIC DL-2000, São José dos Pinhais, PR, Brazil) and subjected to the test (n= 15) at a crosshead speed of 1 mm/min. A stainless-steel rod (Ø= 0.2 mm) was placed on the adhesive interface to ensure that the shear force was applied as close as possible to the bonding surface. Load application continued until debonding of the cement occurred.

The bond strength (MPa) was calculated using the equation S= L/A, where*S*is the bond strength,*L*is the load at failure (N), and*A*is the adhesive interface area (1 mm²). Specimens that exhibited pre-test failures (PTF) after aging were replaced.

### Complementary analysis

After the *µSBS test*, all specimens were examined using a Dino-Lite digital microscope (AnMo Electronics Corporation, Taiwan). Failure modes were classified as predominantly adhesive when more than 50% of the bonded area showed debonding at the resin cement-zirconia interface, or predominantly cohesive when more than 50% of the failure occurred within the resin cement or zirconia material ^46^ ^,^ ^47^. Representative images of each failure type were obtained using scanning electron microscopy (SEM) (Vega3, Tescan) at 150× magnification.

Representative specimens from each group were prepared for detailed analysis and subsequently coated with a thin layer of gold-palladium alloy to enable SEM visualization (Vega3, Tescan, Czech Republic). These specimens were examined at 1,000× magnification to assess the topographical changes resulting from the different cleaning protocols. Additionally, the same specimens were analyzed by energy-dispersive X-ray spectroscopy (EDS) (Vega3, Tescan) to qualitatively identify the chemical elements present after each treatment, with emphasis on detecting silica oxide from the silica-coated procedure, thereby providing information on the elemental composition of the treated zirconia surfaces.

### Data analysis

The statistical power of the study was determined a posteriori based on the observed effect size (d= 0.93), considering all experimental groups, including both baseline and aging conditions from the µSBS test. The total sample analyzed was 120 specimens (15 per group), and the significance level was set at α=. 0.05. The calculation was performed using G*Power 3.1, allowing the estimation of the actual power (1−β) of the study to detect statistically significant differences in bond strength among the evaluated treatments.

Data from the µSBS test were analyzed using SPSS software (Version 26, IBM Corp., Armonk, NY, USA). A Two-way ANOVA was used to evaluate the effects of aging (baseline vs. aging), cleaning protocol (AJ, AWS, UB-A, UB-W) and their interaction on bond strength. Homogeneity of variances was assessed using Levene’s test. Tukey’s HSD was applied for post hoc comparisons, with the significance level set at α= 0.05.

Additionally, the topographical analysis and failure mode classification were conducted qualitatively and descriptively, while the EDS analysis was performed in a semi-quantitative manner, allowing comparison among the different cleaning protocols.

## Results

Based on the observed effect size (d= 0.93) and the total sample size (n= 120, 15 per group), the study achieved a statistical power of 100% to detect significant differences in bond strength between the groups, considering both baseline and aging conditions.

The Two-way ANOVA considering the factors cleaning protocol, aging, and their interaction (cleaning protocol × aging) revealed no statistically significant differences among groups. No effect was detected for cleaning protocol (F = 0.383, p = 0.765), aging (F = 3.196, p = 0.077), or their interaction (F = 0.443, p = 0.723). Assumption testing confirmed that the data met the criterion of homogeneity of variances (F = 1.347, df = 9, 140, p = 0.218). Therefore, variance was equal across groups, supporting the adequacy of the ANOVA model. Post hoc Tukey HSD analysis reinforced the absence of differences among cleaning protocols, with all pairwise comparisons presenting non-significant p-values: AJ vs AWS (p= 0.713), AJ vs UB-A (p= 0.976), AJ vs UB-W (p= 0.514), AWS vs UB-A (p= 0.999), AWS vs UB-W (p= 0.936), and UB-A vs UB-W (p= 0.990). The estimated marginal means of bond strength for each cleaning protocol, before and after artificial aging, are presented in Figure 2, demonstrating that no statistically significant differences were observed among the groups. These results indicate that all groups produced comparable outcomes.

**Figure 2:**
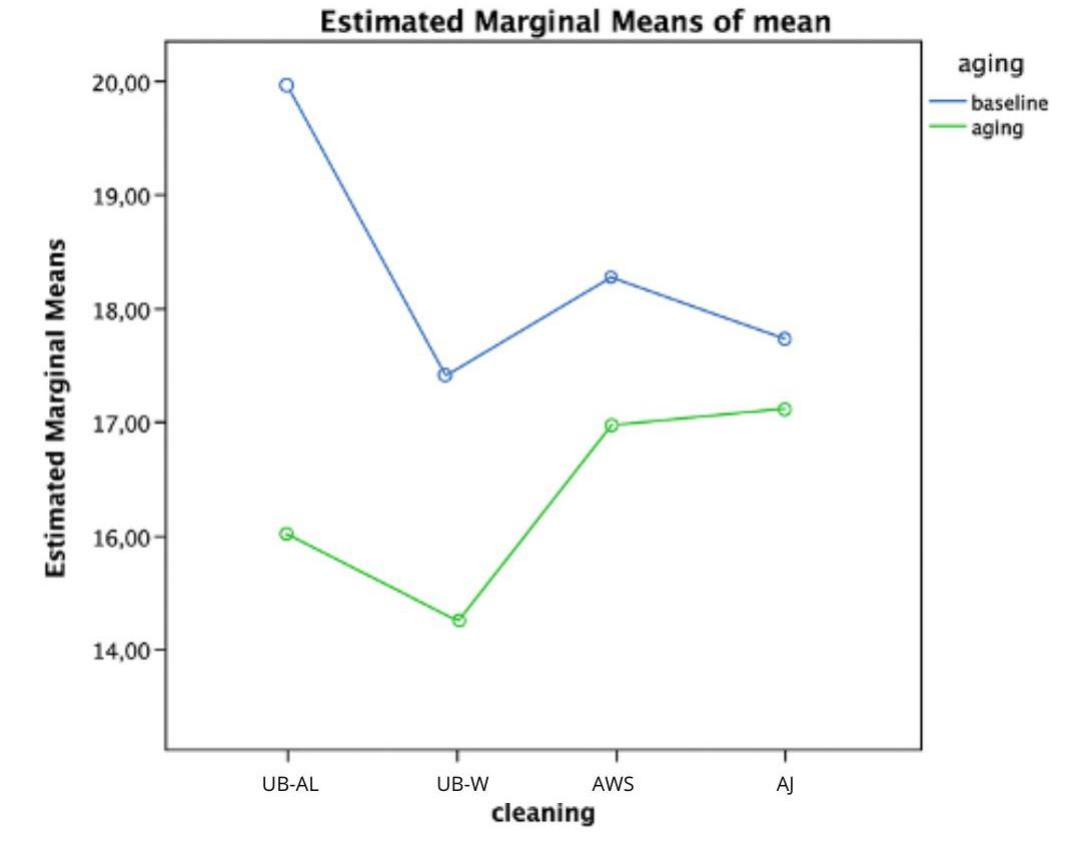
Estimated marginal means of the bond strength data for the different cleaning protocols under baseline and aging conditions.

The topographical assessment by SEM showed that all surfaces exhibited a similar abrasive pattern characteristic of air abrasion. In all conditions, the zirconia surfaces displayed the typical post-air abrasion morphology, marked by micro-irregularities, grooves, and scattered impact sites produced by the abrasive particles (Figure 3). Qualitative EDS analysis showed that the AJ group contained 0.8% silicon, while the AWS group showed 0.13%. The UB-A group, which employed an ultrasonic bath, exhibited the lowest silicon content (0.07%), and no silica was detected in the UB-Wgroup. All groups displayed intense zirconium and oxygen peaks, characteristic of the zirconia substrate (Table 2).

**Figure 3:**
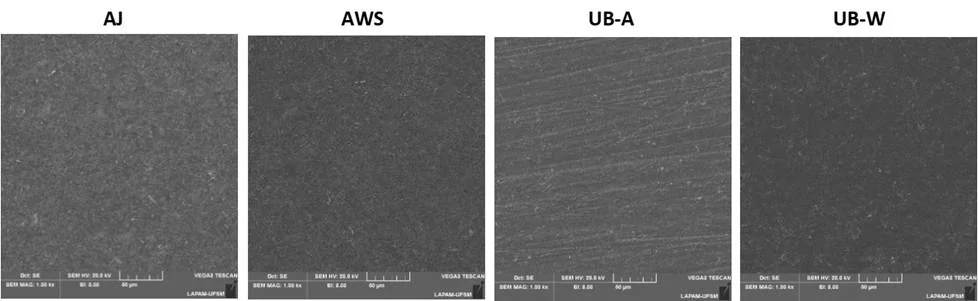
Representative scanning electron microscopy (SEM) images showing the surface topography of the groups at 1,000x magnification.

**Table 2:** Elemental composition (weight %) of the groups obtained by Energy-Dispersive X-ray Spectroscopy (EDS) analysis, showing the weight percentage of Al, Si, and Zr

Group	Al (Alumina)	Si (Silica)	Zr (Zirconia)	O	C
AJ	1.45	0.80	46.44	44.96	6.34
AWS	0.39	0.13	54.56	37.75	7.17
UB-A	0.46	0.07	52.55	40.44	14.75
UB-W	0.30	-	53.15	37.49	0.06

Adhesive and cohesive failures were observed among the groups, as detailed in Tables 2 and 3, and representative images of each failure pattern are presented in Figure 4.

**Table 3:** Results from the microshear test, showing the bond strength mean and standard deviation, as well as the % decrease between Baseline and Aging means for each cleaning protocol, the amount of pre-test failures (PTF), and the characterization of failure pattern (adhesive or cohesive failure).

Group	Storage condition	Bond Strength*	% decrease between Baseline/Aging	PTF (count)**	Failure pattern (n= 15)	
		Mean (SD)			Adhesive (count)	Cohesive (count)
AJ	Baseline	17.96 (7.46)^A^	7.83	-	14	1
	Aging	16.56 (7.72)^A^		6	12	3
AWS	Baseline	17.40 (8.27)^A^	3.93	-	13	2
	Aging	16.72 (7.24)^A^		3	14	1
UB-A	Baseline	19.92 (4.95)^A^	22.70	-	13	2
	Aging	15.40 (6.33)^A^		4	13	2
UB-W	Baseline	17.03 (5.00)^A^	13.80	-	13	2
	Aging	14.68 (7.18)^A^		11	13	2

*Two-way ANOVA considering the factors ‘cleaning protocol’, ‘aging’, and their interaction, cleaning protocol *aging’, showed absence of statistical differences among groups, with F= 0.383 p= 0.765; F= 3.196 p= 0.077; and F= 0.443 p= 0.723; respectively.

** Pre-test failures were detected during the aging process (thermocycling), and such specimens were replaced, resulting in a potential overestimation of the mean of aged groups.

**Figure 4:**
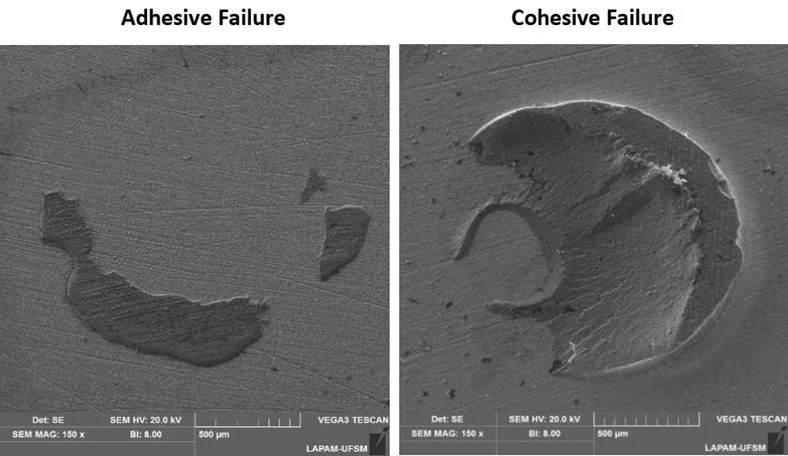
Representative scanning electron microscopy (SEM) images of microshear-tested specimens showing defined failure patterns under 150× magnification.

## Discussion

The present study aimed to assess whether the cleaning protocol of the bonding surface of translucent zirconia, after air abrasion with silica-coated alumina particles, affects its bond strength to resin cement before and after long-term aging. The results supported the null hypothesis, suggesting that the evaluated cleaning protocol did not negatively affect bond strength.

Although variations in residual silica content among the silica-coated groups were observed, these variations did not result in differences in bond strength in translucent zirconia. (Table 3). This indicates that, under the conditions evaluated, the differences introduced by the cleaning protocols were insufficient to affect bond strength. In a study comparing the adhesive performance of 3Y-TZP and 4-YSZ, Minh Le et al. ^48^ reported no differences in bond strength, demonstrating that air abrasion with aluminum oxide combined with MDP-containing cement ensured stability in both groups even after aging. It should be noted that the present study was conducted exclusively with 4-YSZ, which presents a distinct microstructure compared to conventional 3Y-TZP. Therefore, although the material’s characteristics may have influenced its response to surface treatments and cleaning protocol, this relationship cannot be definitively established in the absence of a direct comparison. Future studies comparing different zirconia compositions are necessary to better understand the role of microstructure in the bonding process.

The silica coating on the zirconia surface is essential to promote stable chemical bonds via siloxane bonds between the silane and the resin matrix ^23^ ^,^ ^49^ ^,^ ^50^ ^,^ ^51^. In systems containing 10-methacryloyloxydecyl dihydrogen phosphate (MDP), a dual chemical interface is formed: while silica provides reactive sites for the silane, MDP interacts directly with zirconia, contributing to the initial bond strength ^24^ ^,^ ^36^ ^,^ ^51^. In this manner, the MDP interacts directly with zirconia via its phosphate functional group, forming hydrogen or ionic bonds with zirconium oxide and enabling copolymerization through its methacrylate group ^23^ ^,^ ^48^ ^,^ ^52^ ^,^ ^53^. In our study, no statistically significant differences were observed between baseline and after aging. Similar findings were reported by Minh Le et al. ^48^, who demonstrated that the stability of zirconia bonding is highly dependent on the combination of mechanical surface treatment and adhesive chemistry; groups subjected to air abrasion with aluminum oxide and cement containing MDP exhibited stable bond strength, with no significant differences between baseline and post-aging. Accordingly, the combination of air abrasion and MDP-containing primers is widely accepted for bonding to zirconia ^23^ ^,^ ^48^ ^,^ ^52^ ^,^ ^53^. In this context, our study confirms the effectiveness of this strategy and reinforces the critical role of MDP in achieving initial bond strength and maintaining interfacial stability.

Surface characterization by SEM confirmed that all specimens exhibited the characteristic abrasive pattern of silica-coated air abrasion, with no visible differences among the cleaning protocols (Figure 3). Post-abrasion cleaning is considered a relevant step because it can directly influence the surface energy and wettability of zirconia, which are key factors for bonding. As highlighted by Tzanakakis et al. ^29^, residual particles after air abrasion can compromise these properties, emphasizing the need for methods capable of removing particles without damaging the treated surface. Some studies indicate that ultrasonic baths of substrates abraded with silica can enhance bonding ^54^. In contrast, simple methods, such as air-jet cleaning, are recommended by manufacturers and effectively preserve the silica layer. In the present study, however, all cleaning protocols showed similar bond strength, as demonstrated in Table 3, indicating that none of the approaches provided a relevant advantage for bond strength.

EDS analysis revealed differences in the amount of residual silica among the cleaning protocols. The AJ and AWS groups presented approximately 0.8% and 0.13% silica, respectively, indicating that part of the silica applied during silica-coated air abrasion remained adhered to the surface. In contrast, UB-A and UB-W groups exhibited very low or almost absent silica content, showing that the silica layer was largely reduced after these procedures. Lorente et al. ^36^ reported similar behavior when evaluating ultrasonic cleaning, with a reduction in silica content of 30% after processing. Another study ^35^ also demonstrated that an ultrasonic bath removes silica deposited from the abraded surfaces. In the present study, the UB-A group, which employed an ultrasonic bath, showed the lowest silica content (0.07%), while no silica was detected in the UB-W group, indicating a more aggressive removal of the silica layer. Despite these chemical differences, all protocols resulted in equivalent bonding performance, suggesting that, under the conditions of this study, the amount of residual silica was not a determining factor for bond strength.

Artificial long-term aging reduced bond strength in all groups, which is an expected effect due to the bond degradation promoted by thermocycling and water storage ^15^ ^,^ ^22^ ^,^ ^27^ ^,^ ^49^. Although the means of baseline and aging conditions (Figure 2) had no statistically significant differences, the aged groups exhibited substantial percentages of PTF, characterized by spontaneous debonding during thermocycling (Table 3). As these specimens were replaced, the strategy allowed estimating the bond strength achieved by each protocol but may also have overestimated performance, since the high PTF rate indicates bonding degradation that is not reflected in the final bond strength values. SEM analysis showed that all methods produced clean surfaces, and considering that air and air-water jets are much simpler and less costly alternatives than ultrasound, the morphological similarity among the protocols suggests that conventional approaches may be sufficient to remove post-abrasion residues. Furthermore, the highest percentage of PTF was observed in the UB-W group, indicating that despite the complexity of the method, no greater interfacial stability was achieved after aging. These findings reinforce that cleaning efficiency in such scenario does not necessarily translate into better adhesive performance under accelerated degradation conditions.

Based on these findings, it is possible to answer the research question: after air abrasion with silica-coated alumina particles, which cleaning protocol best maintains bond strength? From the conditions of the present study, all protocols proved safe and capable of preserving bond strength, both immediately and after aging. Additionally, more complex procedures, such as the ultrasonic bath, did not demonstrate additional benefits, contrasting with previous findings ^54^. The ultrasonic bath with distilled water, in particular, showed the highest PTF rates, with no advantages in preserving the silica layer or bond strength (Table 3). Thus, simple methods such as air or air-water jets are viable, fast, low-cost, and clinically efficient alternatives.

Some limitations should be acknowledged. This is an in vitro study conducted under controlled conditions, which do not reproduce important clinical variables such as saliva contamination, moisture variation, or differences in the abrasion pattern. A further limitation is that only one specimen was analyzed by EDS; moreover, as the sole analytical technique employed, EDS has intrinsic limitations related to sensitivity and superficiality, which may not reflect the total uniformity of the silica layer. Furthermore, only four cleaning protocols were evaluated, excluding possible variations in time, pressure, or solvents. Despite these limitations, the results provide relevant evidence concerning the effect of cleaning protocol on the chemical and mechanical bonding of zirconia, reinforcing the need for complementary studies, especially those that more closely simulate the oral environment. An important limitation of this study is the use of a primer containing both MDP and silane (Monobond Plus, Ivoclar AG). Even though the ultrasonic bath likely removed most of the silica deposited on the zirconia surface, the silane component may not have performed effectively. However, the presence of MDP could have compensated for this limitation by enhancing the chemical interaction with the metallic oxides of zirconia. As a result, differences in silica content among the surface treatments may not have substantially influenced the bonding performance, which could help explain the similarity observed between the groups. Although this does not fully account for the comparable results between the baseline and aging conditions, it likely contributed to reducing the expected distinctions.

Finally, based on the results, it can be concluded that, after silica-coated air abrasion, the different cleaning protocols evaluated did not significantly affect the bond strength to translucent zirconia, indicating that simple air-jet cleaning is sufficient to provide and maintain adhesive performance. In addition, EDS analyses demonstrated that the air-jet-only cleaning protocol best preserved surface silica, whereas cleaning protocols involving water or ultrasonic baths resulted in detectable silica loss.

## Data Availability

The research data are available upon request.
